# Giant nonfunctioning adrenocortical carcinoma: a case report and review of the literature

**DOI:** 10.1186/1756-0500-7-769

**Published:** 2014-10-31

**Authors:** Ahmad Almarzouq, Sami Asfar, Sundus Hussain, Adel Al-Hunayan, Saad Aldousari

**Affiliations:** Urology Unit, Department of Surgery, Mubarak Alkabeer Hospital, Kuwait City, Kuwait; Department of Surgery, Faculty of Medicine, Kuwait University, Kuwait City, Kuwait; Department of Pathology, Mubarak Alkabeer Hospital, Kuwait City, Kuwait; Urology Unit, Department of Surgery, Faculty of Medicine, Kuwait University, PO Box 24923, Safat, 13110 Kuwait

**Keywords:** Adrenocortical carcinoma, Adrenocortical mass, Giant, Nonfunctioning

## Abstract

**Background:**

Adrenocortical carcinoma is a rare and aggressive malignancy. Patients usually present early with manifestation of abnormal hormone secretion. However, adrenocortical carcinoma can also be nonfunctioning, and such patients present late with a mass effect or distant metastases.

**Case presentation:**

We herein report a case of a 30-year-old Sri-Lankan woman who presented with a 3-month history of left flank pain associated with nausea, vomiting, and weight loss. Imaging revealed a large left upper quadrant mass with a 1.8-cm left lung nodule. The differential diagnoses included a left adrenal mass, left upper pole renal mass, and retroperitoneal sarcoma. A functional adrenal work-up revealed no abnormal findings. Surgical excision of the mass was uneventful with no postoperative complications. Pathological analysis revealed a nonfunctioning adrenocortical carcinoma measuring 16 × 14 × 10 cm. To our knowledge, a mass of this size is among the largest nonfunctioning adrenocortical carcinomas reported in the published literature. The investigations and approach to treatment were consistent with those in the published literature.

**Conclusion:**

Large nonfunctioning adrenocortical carcinomas pose a diagnostic and therapeutic challenge, and most are diagnosed at a late stage. Appropriate imaging and functional work-up of the mass are vital before treatment. Surgical excision is safe, even for large adrenocortical carcinomas; excision in patients with advanced disease has been shown to have the best outcomes.

## Background

Adrenocortical carcinoma (ACC) is a rare and aggressive malignancy with an estimated annual incidence of one to two cases per million inhabitants in the United States [1, 2]. The age distribution is bimodal, with peaks occurring at 5 to 20 and 40 to 50 years of age [1–3]. A slight female predominance has been shown [1].

Adrenal masses can be broadly divided into hyperfunctioning (hormone-secreting) and nonfunctioning (non-hormone-secreting). Functioning ACC presents earlier with hormonal manifestations such as virilization, feminization, or Cushing’s syndrome. However, nonfunctioning tumors pose a diagnostic challenge because they are diagnosed incidentally due to a mass effect or metastatic disease. Moreover, successful radical management is seldom achievable because most diagnoses are made when the tumor has either invaded local structures or metastasized, explaining the poor prognosis associated with these masses [2, 3].

Even after a grossly complete resection, most patients develop early tumor recurrence or distant metastasis [2]. This is presumed to be due to occult micrometastases that were not detected at the time of diagnosis [4, 5]. The 5-year overall survival rate ranges from 16% to 44% [6]. However, a more recent case series suggested an improved 5-year overall survival rate reaching 60%; in this study, 34% of the enrolled patients had metastatic disease at diagnosis [6]. Early-stage disease in the absence of lymph node and distant metastases and an age of <40 years have been shown to be positive prognostic factors [7, 8]. Additionally, negative resection margins have been shown to improve survival independent of other factors [9].

The most frequent metastatic sites are the lung, lymph nodes, liver, bone, and inferior vena cava at 71%, 68%, 42%, 26%, and 20%, respectively [10]. We herein present a case of a giant nonfunctioning ACC with local tumor effects.

## Case presentation

A 30-year-old Sri-Lankan woman presented with left flank pain. The pain was intermittent, sharp, and of moderate severity and radiated to the groin. The patient reported no history of lower urinary tract symptoms or gross hematuria. The pain was associated with weight loss of 8 kg over a period of 3 months. Her medical and surgical history was not significant, and the patient was not on any regular medications.

**Figure 1:**
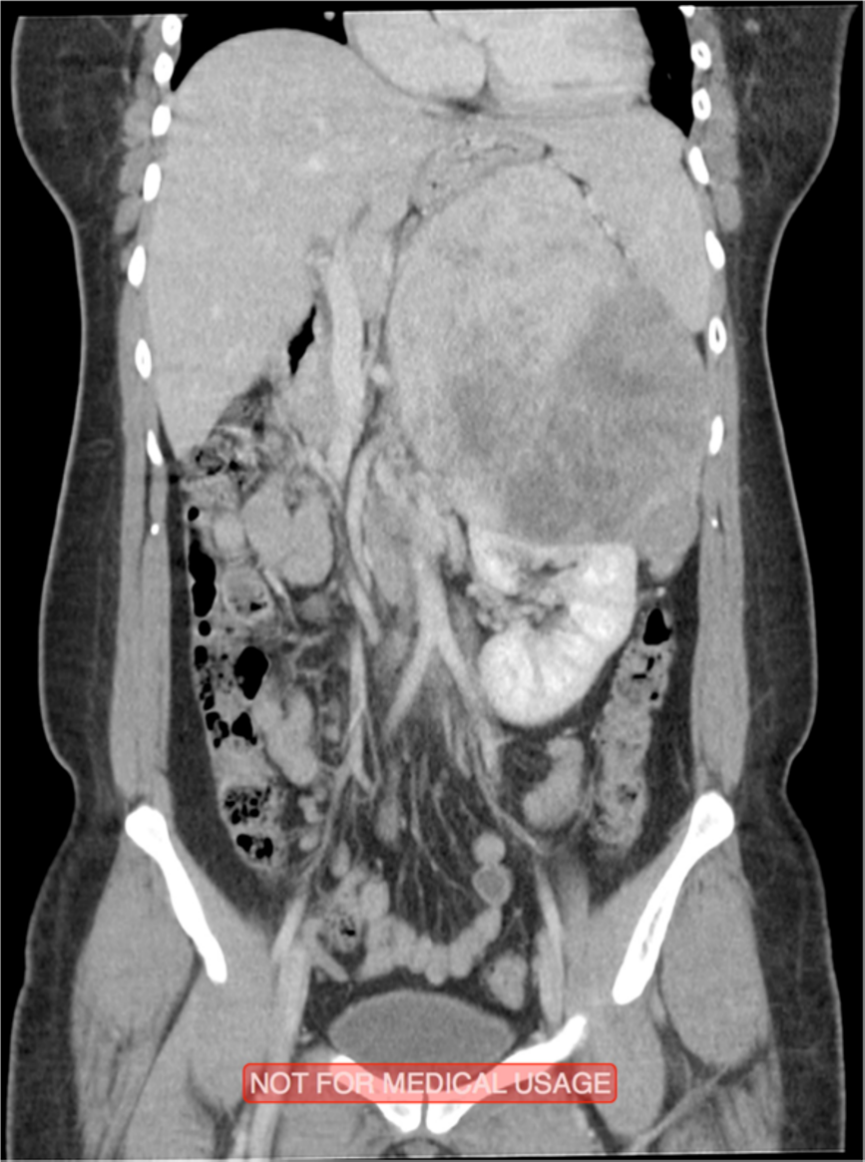
**A coronal section of the abdomen and pelvis illustrating the size of the mass.** The mass is shown compressing and displacing the adjacent structures and occupying the whole left upper quadrant.

**Figure 2:**
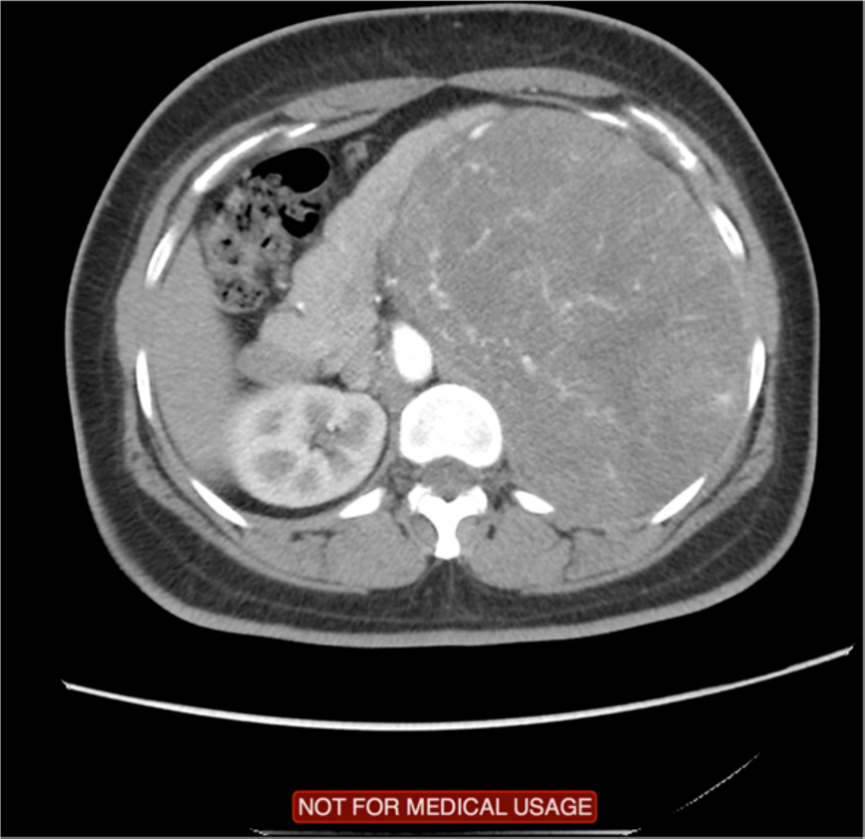
**A cross section of the abdomen showing the large mass crossing the midline.**

The patient was afebrile on presentation with no microscopic hematuria. On physical examination, the abdomen was soft with minimal tenderness over the left flank, and a palpable left upper quadrant mass was present. The rest of the clinical examination revealed normal findings.A computed tomography (CT) scan showed a large left upper quadrant mass measuring 17 × 13 × 12 cm in its greatest dimension. The mass crossed the midline and compressed the stomach, spleen, pancreas, and small bowel (Figures 1 and 2). The origin of the mass was unclear, and the differential diagnoses included a left adrenal mass, left upper pole renal mass, and retroperitoneal sarcoma. A functional adrenal work-up was performed according to the Canadian Urological Association guidelines and included measurement of serum aldosterone, potassium, renin, and adrenocorticotrophic hormone levels; a dexamethasone suppression test; and measurement of 24-hour urinary metanephrine levels. All results were within the reference ranges.

The metastatic work-up included CT scans of the head and chest and a bone scan. The head CT scan revealed normal findings. The chest CT scan showed a 1.8-cm nodule in the medial left lower lobe, close to the posterior mediastinum, as well as two subcentimetric nodules in the left lung. Neither the bone scan nor magnetic resonance imaging (MRI) showed evidence of bone metastasis. A decision was made to proceed with en bloc excision of the mass. Cardiothoracic surgeons were consulted regarding the suspicious lung nodules, and they planned to biopsy the lung lesion after excision of the primary tumor.

### Operative findings

A Chevron incision was made, and a Gomez poly tract retractor (Pilling**®**) was used to ensure adequate exposure. The mass was exposed after medial reflection of the descending colon and was found to be displacing the stomach, pancreas, and spleen, but it had a grossly negative margin with no evidence of adjacent organ invasion. The mass was covering the left renal hilum and was densely adhered to the aorta. After adequate exposure of the left kidney, the mass was noted to be densely adhered to the upper pole with no surgically defined plane. It seemed to be arising from the upper pole of the left kidney. In the presence of a vascular surgeon, the mass was dissected and the left renal hilum was carefully isolated. We decided intraoperatively to proceed with left radical nephrectomy. This decision was made to avoid positive surgical margins if partial nephrectomy was performed given the wide base of tumor adherence to the kidney. Upon vascular control of the left renal artery and vein, the mass significantly shrunk in size and became less congested. It was then easily dissected off the aorta. No enlarged lymph nodes were identified. After ensuring adequate hemostasis, Surgicel® Fibrillar™ was applied, and an intra-abdominal drain was inserted.

The postoperative recovery was uneventful. The drain was removed on postoperative day 2, and the patient was discharged on postoperative day 6. She was followed up 4 weeks postoperatively with no complications. After returning to her home country, her local cardiothoracic surgeon obtained biopsy specimens from the lung nodules; these were found to be benign granulomas. However, contrast-enhanced CT of the chest, abdomen, and pelvis performed 3 months postoperatively showed new liver metastasis.

### Histological findings

**Figure 3:**
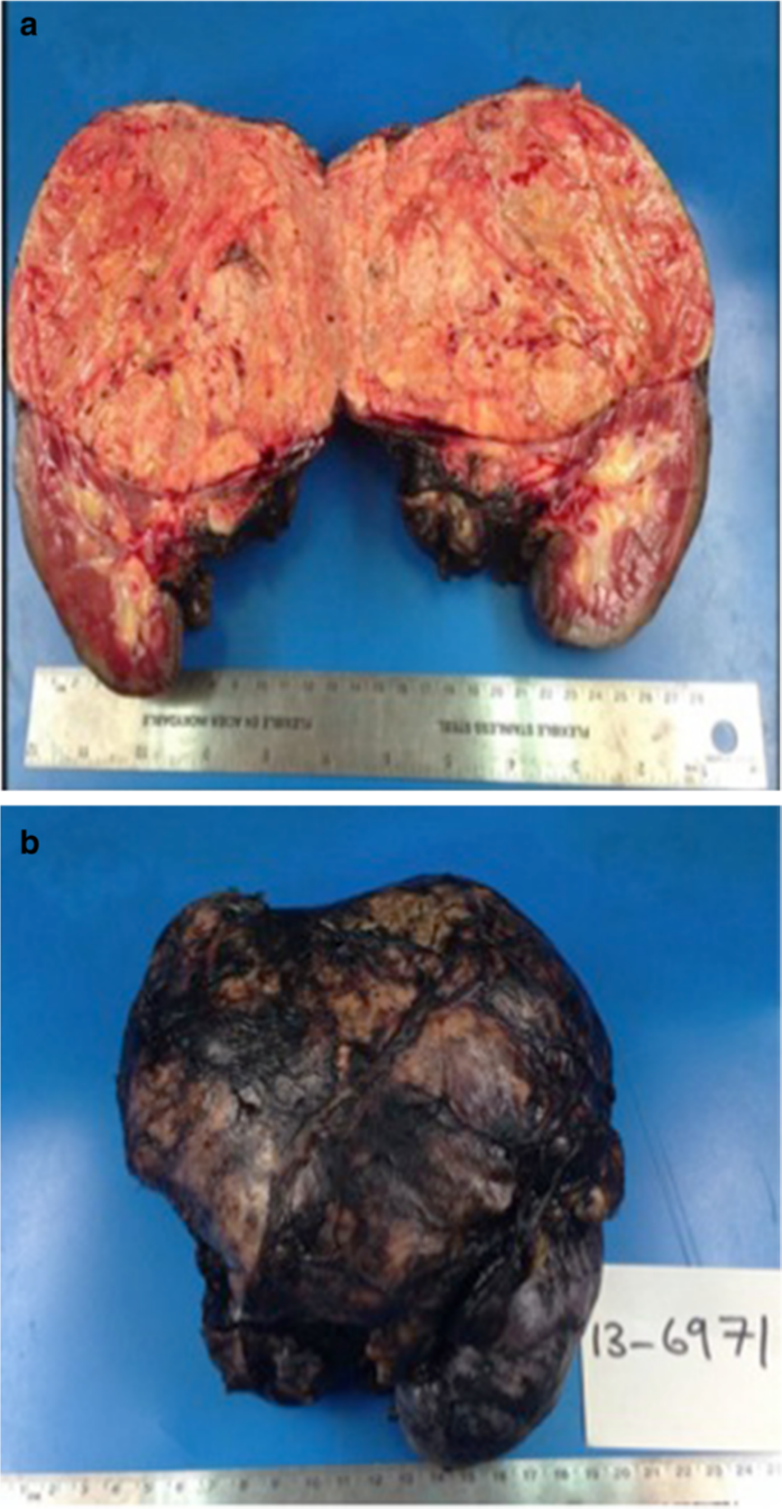
**Illustrates the extracted mass which includes the kidney and the adrenal gland. a)** Gross appearance of the specimen showing a well-encapsulated mass with a distinct plane separating it from the kidney. **b)** En bloc specimen including the left kidney.

**Figure 4:**
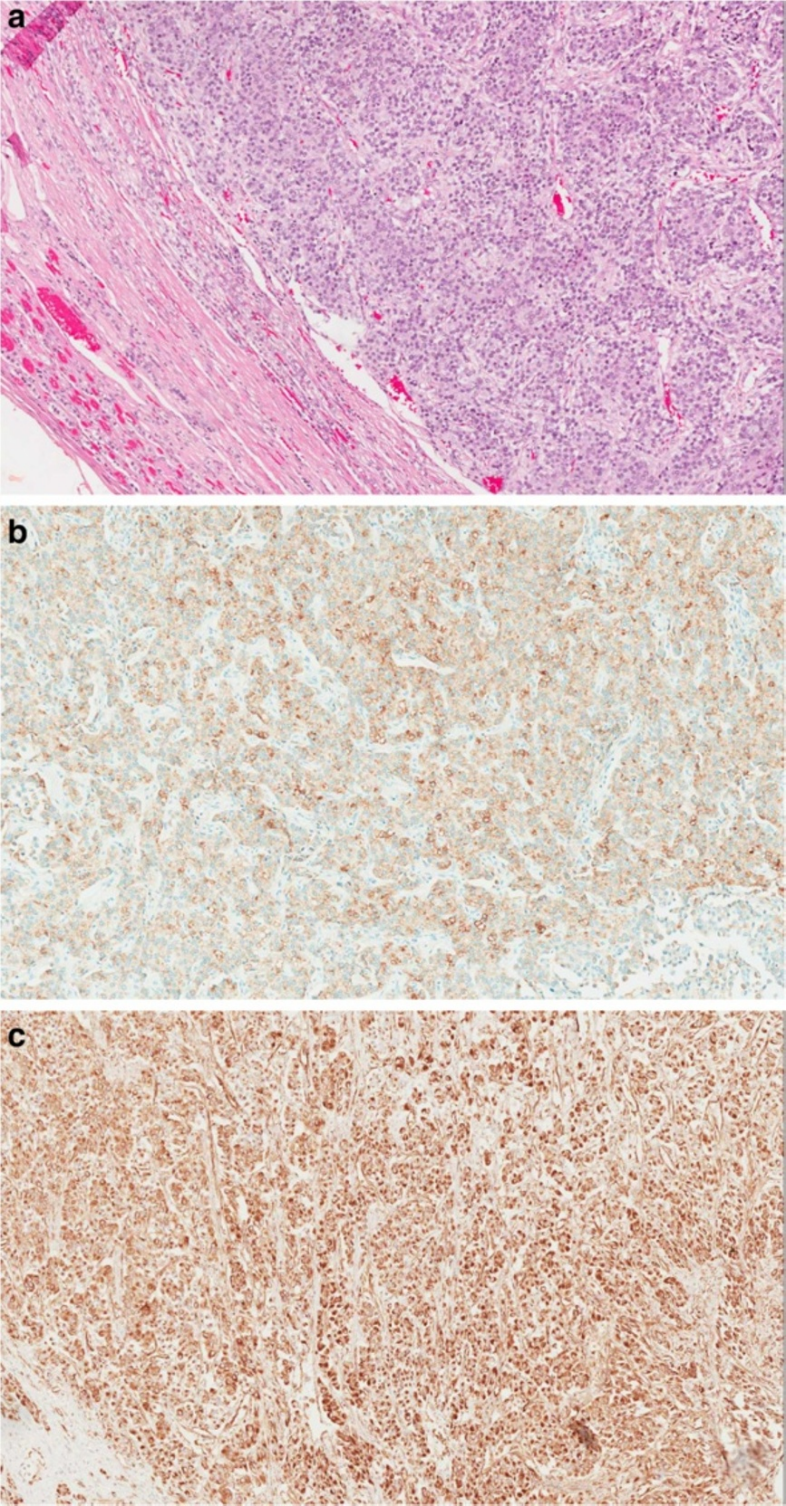
**Illustrates the histopathological and immuno-cytochemistry stained slides as described in a, b and c. a)** Hematoxylin and eosin stain of the adrenal gland. Right: Tumor. Left: Residual rim of normal adrenal gland (10×). **b)** Immunohistochemical stain: inhibin (10×). **c)** Immunohistochemical stain: vimentin (10×).

Histological examination showed a 1.63-kg en bloc left radical nephrectomy specimen that measured 20.0- × 16.5- × 11.0-cm (Figure 3a and b). Opening of the specimen revealed a huge mass arising from the left adrenal gland measuring 16 × 14 × 10 cm. The tumor was tan-yellow and well demarcated, and it showed a clear line of cleavage from the left kidney. Histological examination of the tumor showed overt features of malignancy including a high nuclear grade, high mitotic activity (>10/50 high-power fields), and foci of diffuse growth admixed with single infiltrating cells. Venous and capsular invasion was also present. Immunohistochemistry revealed positive staining of tumor cells for synaptophysin and inhibin, supporting a diagnosis of ACC (Figure 4a–c). The margins were uninvolved by the tumor. In accordance with the 2010 Tumor Nodes Metastasis staging system, the tumor stage was pT3NxMx.

## Conclusions

Approximately 60% of patients with ACC present with symptoms and signs of hormonal secretion. This was not a feature in the present case. Hormone secretion is not a discriminating feature between benign and malignant adrenocortical masses. The silent clinical nature of nonfunctioning ACC results in poorer outcomes. Hence, the majority of patients with nonfunctioning ACC present with locally advanced and/or metastatic disease. The work-up for adrenal masses must include determination of whether the mass is functioning or nonfunctioning and whether it is benign or malignant. Hormonal evaluation helps define the functional status of the tumor and can also help define its origin. Radiographic studies in the form of CT or MRI can help define the size of the mass and rule out metastases. Emerging evidence suggests that fluorodeoxyglucose-fused positron emission tomography with CT (FDG-PET/CT) is superior to CT alone [11]. However, FDG-PET/CT is still considered a complimentary study and is not recommended for ACC work-up [11].

With the presence of a questionable metastatic pulmonary nodule and large tumor of >4 cm, the clinical suspicion of malignancy was high in this case and favored radical adrenalectomy [12–14]. To the best of our knowledge, a mass of this size is among the largest nonfunctioning ACCs reported in the literature [15, 16].

Despite the poor prognosis in this patient group, chemotherapy has a limited role in the treatment of ACC, and surgical resection has been shown to have the best outcomes [7, 8]. However, recent studies have shown a role for adjuvant chemotherapy in prolonging recurrence-free and overall survival [5, 8, 9]. There is no established duration of adjuvant chemotherapy. High-risk patients are currently being treated for 5 years with mitotane. In patients with proven lung metastasis, the published evidence suggests that en bloc excision of involved organs and pulmonary metastasectomy could improve overall survival [5, 8]. Postoperative surveillance for recurrence should be performed every 3 months for the first 2 years and then every 6 months for 5 years. Contrast-enhanced CT scans of the chest, abdomen, and pelvis are the standard of care for follow-up; however, FDG-PET/CT scans are used in some specialized centers and are still under investigation [11]. We have herein illustrated the successful and safe surgical resection of a large ACC with no postoperative complications. We will provide an update on the condition and survival of the patient when available.

## Consent

Written informed consent was obtained from the patient for publication of this Case Report and any accompanying images. A copy of the written consent is available for review by the Editor-in-Chief of this journal.

## Abbreviations

ACC: Adrenocortical carcinoma
CT: Computed tomography
MRI: Magnetic resonance imaging
FDG: Fluorodeoxyglucose
PET: Positron emission tomography.

## References

[1] Norton JA, DeVita VTJr, Hellman S, Rosenberg SA (2005). Adrenal tumors. Cancer: Principles and Practice of Oncology.

[2] Icard P, Goudet P, Charpenay C, Andreassian B, Carnaille B, Chapuis Y, Cougard P, Henry JF, Proye C (2001). Adrenocortical carcinomas: Surgical trends and results of a 253-patient series from the French Association of Endocrine Surgeons Study Group. World J Surg.

[3] Kopf D, Goretzki PE, Lehnert H (2001). Clinical management of malignant adrenal tumors. J Canc Res Clin Oncol.

[4] Gaujoux S, Al-Ahmadie H, Allen PJ, Gonen M, Shia J, Dángelica M, Dematteo R, Fong Y, Jamagin WR (2012). Resection of adrenocortical carcinoma liver metastasis: is it justified?. Ann Surg Oncol.

[5] Kemp C, Ripley R, Mathur A, Steinburg SM, Nguyen DM, Fojo T, Schrump DS (2011). Pulmonary Resection for Metastatic Adrenocortical Carcinoma: The National Cancer Institute Experience. Ann Thorac Surg.

[6] Vassilopoulou-Sellin R, Schultz PN (2001). Adrenocortical carcinoma. Clinical outcome at the end of the 20th century. Cancer.

[7] Ayala-Ramirez M, Jasim S, Feng L, Ejaz S, Deniz F, Busaidy N, Waguespack SG, Naing A, Sircar K, Wood CG, Pagliaro L, Jimenez C, Vassilopoulou-Sellin R, Hebra MA (2013). Adrenocortical Carcinoma: Clinical Outcomes and prognosis of 330 Patients at a Tertiary Care Center. Eur J Endocrinol.

[8] Schulick RD, Brennan MF (1999). Long-Term Survival After Complete Resection and Repeat Resection in Patients With Adrenocortical Carcinoma. Ann Surg Oncol.

[9] Schteingart DE, Doherty GM, Gauger PG, Giordano TJ, Hammer GD, Korobkin M, Worden FP (2005). Management of patients with adrenal cancer: recommendations of an international consensus conference. Endocr Relat Cancer.

[10] Norton J, Le H, DeVita VT (2001). Adrenal tumors. Cancer.

[11] Deandreis D, Leboulleux S, Caramella C, Schlumberger M, Baudin E (2011). FDG PET in the management of patients with adrenal masses and adrenocortical carcinoma. Horm Cancer.

[12] Weiss LM (1984). Comperative histologic study of 43 metastasizing and non metastasizing adrenocortical tumors. Am J Surg Pathol.

[13] Tseng Y (2013). Wu s, Chao, Wu C J, Chau T.

[14] Kapoor A, Morris T, Rebello R (2011). Guidelines for the Management of the Incidentally Discovered Adrenal Mass. Can Urol Assoc J.

[15] National Institutes of Health (2002). “NIH state-of-the-science statement on management of the clinically inapparent adrenal mass (“incidentaloma”). NIH Consensus and State-of-the-Science Statements.

[16] Benassai G, Desiato V, Bianco T, Seviro L, Compagna R, Vigliotti G, Limite B, Quatro G (2014). Adrenocortical carcinoma: What the surgeon needs to know. Case report and literature review. Int J Surg.

